# Illness causal beliefs in Turkish immigrants

**DOI:** 10.1186/1471-244X-7-34

**Published:** 2007-07-24

**Authors:** Harry Minas, Steven Klimidis, Can Tuncer

**Affiliations:** 1 Centre for International Mental Health, School of Population Health, The University of Melbourne, Parkville, Australia; 2 Victorian Transcultural Psychiatry Unit, St Vincent's Health Melbourne, Fitzroy, Australia

## Abstract

**Background:**

People hold a wide variety of beliefs concerning the causes of illness. Such beliefs vary across cultures and, among immigrants, may be influenced by many factors, including level of acculturation, gender, level of education, and experience of illness and treatment. This study examines illness causal beliefs in Turkish-immigrants in Australia.

**Methods:**

Causal beliefs about somatic and mental illness were examined in a sample of 444 members of the Turkish population of Melbourne. The socio-demographic characteristics of the sample were broadly similar to those of the Melbourne Turkish community. Five issues were examined: the structure of causal beliefs; the relative frequency of natural, supernatural and metaphysical beliefs; ascription of somatic, mental, or both somatic and mental conditions to the various causes; the correlations of belief types with socio-demographic, modernizing and acculturation variables; and the relationship between causal beliefs and current illness.

**Results:**

Principal components analysis revealed two broad factors, accounting for 58 percent of the variation in scores on illness belief scales, distinctly interpretable as natural and supernatural beliefs. Second, beliefs in natural causes were more frequent than beliefs in supernatural causes. Third, some causal beliefs were commonly linked to both somatic and mental conditions while others were regarded as more specific to either somatic or mental disorders. Last, there was a range of correlations between endorsement of belief types and factors defining heterogeneity within the community, including with demographic factors, indicators of modernizing and acculturative processes, and the current presence of illness.

**Conclusion:**

Results supported the classification of causal beliefs proposed by Murdock, Wilson & Frederick, with a division into natural and supernatural causes. While belief in natural causes is more common, belief in supernatural causes persists despite modernizing and acculturative influences. Different types of causal beliefs are held in relation to somatic or mental illness, and a variety of apparently logically incompatible beliefs may be concurrently held. Illness causal beliefs are dynamic and are related to demographic, modernizing, and acculturative factors, and to the current presence of illness. Any assumption of uniformity of illness causal beliefs within a community, even one that is relatively culturally homogeneous, is likely to be misleading. A better understanding of the diversity, and determinants, of illness causal beliefs can be of value in improving our understanding of illness experience, the clinical process, and in developing more effective health services and population health strategies.

## Background

Beliefs concerning causes of illness vary considerably across cultures [1-5]. There have been several attempts to organize the wide variety of causal beliefs into a small number of coherent dimensions [6,7]. Murdock et al. [7] drew together causal beliefs from 139 traditional and contemporary societies from the World Ethnographic Atlas, identifying two broad constructs, natural and supernatural, and a variety of sub-constructs within each of these. These included, within the natural group, causes such as stress, infection, and organic deterioration, and, within the supernatural group, causes such as fate, mystical retribution, and magical causation. There is some empirical support [8,9] for distinguishing between the types of causes identified by Murdock et al. [7]. Eisenbruch [2] suggested the addition of non-Western 'physiological' causal constructs that are commonly found in Asian medical traditions (e.g., yin/yang, hot/cold, distribution or flow of energies, ethers or forces, etc.). Multi-dimensional scaling revealed four broad clusters of causes of 'mental distress': somatic/biological (e.g., genetic defect, brain damage, bad nerves); stress (including physical, circumstantial and due to life events); mystical (e.g., violation of a taboo, provocation by a spirit, casting of a spell); and, metaphysical (e.g., movements of wind in the body, out of balance or harmony) [2]. Subsequent factor analytic work in Hong Kong confirmed the validity of this categorisation of causal beliefs in Asian samples [4].

Examinations of more traditional societies suggest that supernatural beliefs are common [e.g. [1,5,10-12]]. In a study of Nigerian psychiatric out-patients approximately half of the participants attributed their illness to supernatural causes [10]. All of the 60 patients in Al-Krenawi's study of Bedouin-Arab psychiatric outpatients in Israel attributed their disorder to supernatural causes [1]. Whether supernatural beliefs persist despite modernization and acculturative influences on immigrants [see, [13]] remains unclear. Belief in supernatural causes is not limited to traditional societies, with such beliefs being endorsed by a substantial proportion of respondents in a sample of university students in the USA [8].

Causal beliefs may be influenced by the type of illness experienced [9,14-17], by gender and by level of education. In the Nigerian sample referred to above [10] women and the more educated more commonly attributed their illness to psychosocial causes rather than to supernatural causes. However, in Al-Krenawi's study [1] causal beliefs were not related to gender, education, social class or type of illness.

While only few studies have examined illness causal beliefs in immigrant or ethnic minority groups [e.g. [2,18,19]] an understanding of causal beliefs is important for the design of culturally appropriate mental health services [20] and for accurate diagnosis and treatment [21].

Illness causal beliefs have been subject to some investigation in Turkey, particularly in relation to to mental disorders [22-29]. Early studies of predominantly rural Turkish populations reported adherence to traditional beliefs and the use of magico-religious therapeutic practices [27-29]. Ozturk [28] reported that traditional beliefs are strongly influenced by Islam, including fatalistic beliefs ('God's will'), specific beliefs regarding harm caused through possession by spiritual beings (jinns) described in the Qur'an, retribution for transgression of religious taboos, and failure to take precautions against jinns. The 'evil eye', in which there was widespread belief, was associated with psychological and physical illness as well as failures in life and personality problems. Sorcery was associated with conditions such as inability to concentrate, physical weakness, agitation, headaches, confusion and emotional distress. However, many natural beliefs also prevailed. Karanci [24] examined the beliefs of psychiatric inpatients in Ankara. Factor analysis of a set of beliefs rated on their relevance to personal illness revealed seven dimensions: interpersonal conflicts, nuclear family conflicts, extended family conflicts, fate and material difficulties, personal characteristics caused by others, lack of willpower, and personal characteristics. Similar results were found in a group of psychiatric outpatients [25] and subsequent analysis revealed tendencies for patients to give priority to beliefs concerning the impact of family conflict, personal characteristics and work-related difficulties. However, this work did not include a comprehensive assessment of supernatural causes.

Australia is a multicultural society with a large proportion of its population made up by immigrant groups. The cultural diversity of the population presents many challenges in the provision of effective and equitable health services [30]. The Turkish community in Australia was the first substantial migration to Australia from a non-Western European country.

In this study we examine the following aspects of illness causal beliefs in Melbourne's Turkish community: the relationships among causal beliefs using exploratory factor analysis, assessing whether two super-ordinate dimensions, natural and supernatural, can be confirmed; the frequency of the various causal beliefs in this community;

the attribution of somatic (S), mental (M), and both mental and somatic (MS) illness, to the various categories of cause; the association of illness causal beliefs with indicators of within-group diversity, including sociodemographic and migration variables; and the association of illness causal beliefs with the current presence of illness.

## Methods

### Sampling

To develop a representative sample, the geographic distribution of the Turkish community in Melbourne (capital city of the state of Victoria) was examined using the Australian Population Census. Sixty-three percent of Victoria's Turkish community resides in the Northern and Western suburbs of Melbourne. The Istanbul telephone directory was used to identify distinctive Turkish names with a high frequency of occurrence. This list was then used to identify households in Melbourne by use of the local telephone directory. Prior to contacting any household, to invite participation in the study, researchers announced the study on Turkish-speaking radio programs and elicited support for the study from many of the Turkish community organizations in Melbourne. Following this a letter, in Turkish and English, was sent to each household informing the occupants that a researcher would make telephone contact within a week to describe the details of the study and to invite participation of an adult household member. For sampling independence, only one adult per household was invited to participate in the interview. For half of the households males were selected and the other half females.

### Questionnaire

The Migration and Settlement Questionnaire (MASQ) and the Explanatory Models of Illness Questionnaire (EMIQ) were used in this work (Minas & Klimidis, *unpublished*, *available on request*). Questionnaires were developed in English and then translated into Turkish using group translation (three bilingual research assistants qualified in sociology and psychology, and one Turkish psychiatrist – CT). Broadly the MASQ covered a wide range of sociodemographic and settlement information including: education; satisfaction with the Australian socio-cultural environment (Cronbach alpha = .77); English language proficiency (Cronbach alpha = .97, [31]); behavioural acculturation (Cronbach alpha = .86); pre-migration rural/urban origin. The measurement domains of the EMIQ include physical and psychiatric symptoms; perceived causes of illness or problems; the impact of illness; and an inquiry into self-help and professional help methods used to resolve illness or problems. In addition, a Turkish version of the GHQ [32] was developed [33] and administered (total scale Cronbach alpha = .97). Causal beliefs were examined by asking 1) whether the respondent had heard of a particular item as a cause of illness (described as 'troubles in the body or emotions or mental life'), and, if so, 2) whether the respondent believed this to be a cause of illness, and, if so, 3) whether the respondent believed that it was a cause somatic (S) illness, mental (M) illness, or both mental and somatic (MS) illness. The causal beliefs instrument (*unpublished*, *available from authors on request*) consisted of 120 items developed from multiple sources, as described in the Introduction.

Due to the length of the overall interview the MASQ and EMIQ and the illness causal beliefs instrument were developed to contain mainly closed-ended item format. The interview was conducted in either English or Turkish depending on the preference of participants.

### Procedure

Interviewers, who were all fluent in Turkish and English, were trained using rehearsal methods in the delivery of the questionnaire and scoring of the questions. A coding instruction booklet was provided for interviewers to help them resolve any coding difficulties. Researchers were available to the interviewers throughout the fieldwork period to clarify any difficulties with interviewing procedures. Once interviewers attended the home at the invitation of potential participants the study was described and a household member was invited to participate. All participants were asked to sign a consent form outlining that they were adequately informed about the study and that they agreed to participate in the study under conditions of anonymity and confidentiality. At the completion of the questionnaire, the consent form was removed from the rest of the questionnaire, separating participant identifying information from his or her responses.

Data were entered into a computer database using a purpose-written program, and checked for entry accuracy. Analyses were conducted using the Statistical Package for the Social Sciences. Specific measures and statistics are described below.

The study was approved by the Victorian Mental Health Research Institute Research and Ethics Committee.

## Results

### Sample representativeness and characteristics

Based on Australian census data, 93% of all Turkish-born people in Victoria live in Melbourne. In the geographic regions in Melbourne (northern and western suburbs) from which we sampled there was a total population of 6,397 Turkish-born people, constituting 53% of all age-eligible Turkish-born living in Melbourne, and 43% of all age-eligible Turkish-born living in Victoria. We interviewed 444 members of the community, with each participant being from a different household (to maintain independence between sampling units for the purposes of analysis). If each household had at least two adults then the number of eligible members of the population was reduced from 6,397 available for sampling to 3,199. On this basis we interviewed approximately 1 in every 7 people (444/3199) available for interview (or 13.9% of the population).

The sample consisted of 213 males and 231 females. Examination of comparative data from the Australian population census revealed that the socio-demographic characteristics of the sample were broadly similar to those of the Melbourne Turkish community (Table 1).

**Table 1:** Population and sample characteristics: representativeness of sample

	**Population**	**Sample**
Female/Male ratio	1.1	0.9
Median age (years)	33	35
Age distribution (%)		
18–24 years	16.7	13.1
25–34 years	34.0	36.0
35–44 year	21.5	22.8
44–54 years	16.9	18.9
>54 years	10.9	9.2
Education: No post-secondary or vocational qualification (%)	74.9	73.1
Unemployment rate (%)	31.2	25.5

The majority of the sample (84 percent) was in a marital relationship and average age was 36.9 years (sd = 11.62). Nearly 46 percent of the sample had primary school education or less, a further 40 percent completed part or all of high school, and 13 percent had progressed beyond high school education. A small group (11 percent) arrived in Australia prior to 1969, half of the group (50 percent) between 1970 and 1979, with the remainder of the group having arrived after 1979. While 71 percent originated from an urban setting in Turkey, 19 percent had resided in rural settings and the remaining 9 percent spent time in both rural and urban settings in the two years prior to migration. Mean satisfaction with life in Australia was 1.6 (sd = .60), reflecting mid-way ratings between 'a little' and 'reasonably' satisfied (on a scale where extreme points were labelled 'not at all' and 'completely'). Mean acculturation level was 2.3 (sd = .64) on a five point scale (1 = only Turkish to 5 = only Australian/English) with low values reflecting preference for Turkish culture. The score of 2.3 reflects the response category of 'mostly Turkish' across the seven items of the scale. Mean English language proficiency was low in the sample with analysis indicating 'a little' to 'fair' ability in communicating in English in simple situations such as shopping and regular banking, and between 'not at all' and 'fair' in more complex situations such as communicating with an English-speaking doctor [31]. In relation to health measures, 53.2 percent indicated tat they were suffering from an illness, either somatic or mental, and 18.2 percent classified themselves as unable to work due to illness or injury (invalid status). Twenty-three percent of the sample met criteria for psychological morbidity according to the GHQ-60 (12 or more symptoms present).

### Factor structure of illness causal beliefs and scale properties

First we sought to test the classification of illness beliefs suggested by Murdock et al [7] by exploring whether their suggested broad classification scheme would emerge through principal components analysis. Table 2 shows the final outcome of principal components analysis on the correlation matrix of the sum of causal beliefs within each scale's item set. Only two factors emerged with eigenvalues of one or more and there was a clear change in the gradient of the 'scree' beyond this point. Oblique rotation was applied to help interpret the factors. First, the solution shows a clear simple structure, and the factors conform to the typological distinction between Natural (Factor 1) and Supernatural (Factor 2), as suggested by Murdock et al. In this sample Metaphysical causes appear to be closely related to Natural causes, with subsequent correlation analysis indicating that they were most strongly correlated with Organic Deterioration (r = .64, df = 443, p < .001) and Physical Stress (r = .55, df = 443, p < .001). The solution accounted for 58.7 percent of the variance in scale scores and most of the scales, with the exception of Contagion, had significant portions of their variance explained (ranging between 41 and 87 percent). The poor of fit of Contagion is probably related to its low reliability (as indicated in Table 2), given also that it is a brief, three-item scale. Correlation between the two factors was .40. As indicated in Table 2 (caption) two broad, reliable scales could be constructed from the items from each factor, separating out Natural and Supernatural causes. Metaphysical causes were kept separate presenting in order to examine their specific associations with other variables. Each scale demonstrated good internal coherence according to the Cronbach alpha statistic (Table 2). Scores on scales were correlated: Natural with Supernatural, r = .43, df = 443, p < .001; Natural with Metaphysical, r = .54, df = 443, p < .001; and Metaphysical with Supernatural, r = .26, df = 443, p < .001.

**Table 2:** Pattern matrix (oblique rotation) of causal belief types based on belief frequency summed within belief types

	**Number of Items**	**Factor 1**	**Factor 2**	**Communality**	**Scale alpha**
*ORGAN: Organic Deterioration	23	**.96**	-.08	.87	.91
*PHTR: Physical Stress	15	**.91**	-.02	.82	.86
*ACCID: Accident Plus	9	**.84**	.03	.74	.73
*PSTR: Psychological Stress	10	**.83**	.10	.76	.82
*INFECT: Infection/Infestation	4	**.72**	-.07	.49	.57
*LITR: Life Events/Circumstances Stress	17	**.70**	.27	.72	.89
META: Metaphysical	5	**.66**	-.02	.43	.84
^†^MAGIC: Animistic/Magical	6	-.08	**.84**	.66	.66
^†^RETN: Non-accidental Mystical Retribution	12	.08	**.77**	.65	.78
^†^OMEN: Ominous Sensations	2	.00	**.72**	.51	.23
^†^FATE*	5	-.06	**.66**	.41	.30
^†^RETA: Accidental Mystical Retribution	5	.00	**.64**	.41	.45
^†^CONTA: Contagion	3	.11	**.38**	.19	.12

%variance		43.3	15.4	Total % var = 58.7	
Factor correlation		.40			

*Natural Beliefs a = .92; ^†^Supernatural a = .75

### Frequency of belief types

Relative frequency of causal beliefs in the sample is summarized in Table 3 which describes the mean percentage of the sample as well as the within belief type percent of sample believing in the belief type. Generally Natural causes were more frequent than Supernatural causes (the former ranging between 30 to 91 percent of the sample and the latter between one and 33 percent of the sample). Metaphysical beliefs were common, ranging between 31 and 58 percent of the sample. The mean percent ranges of the sample believing in the broad types of Natural, Supernatural and Metaphysical beliefs, when these were averaged percentages across specific beliefs, were respectively, 58 to 68, 8 to 22, and, 47 percent.

**Table 3:** Frequency and illness attribution of classes of causes in the Turkish community

				**Mean % classed to cause**		
				
	**Number of Items**	**Mean % of sample believing this**	**Cross-item range of % of sample**	**Somatic Illness (S)**	**Mental Illness (M)**	**Mental & Somatic (MS)**
**NATURAL CAUSATION**						
*Stress*						
INFECT: Infection/Infestation	4	58.3	28.4 – 88.1	69.0	2.0	23.2
PHTR: Physical Stress	15	72.1	28.2 – 91.2	62.8	6.6	27.4
PSTR: Psychological Stress	10	66.0	37.8 – 88.5	13.4	62.9	21.4
LITR: Life Events/Circumstances Stress	17	59.6	27.9 – 76.1	3.7	68.2	25.1
*Organic and Accident*						
ORGAN: Organic Deterioration	23	66.6	21.9 – 76.1	55.5	11.3	29.4
ACCID: Accident Plus (Accidental Causation/Overt Human Aggression/Constitution/Inheritance)	9	67.7	30.4 – 88.5	32.8	14.3	48.8
**SUPERNATURAL CAUSATION**						
*Mystical Causation*						
FATE	5	13.0	1.4 – 33.1	24.3	26.8	39.2
OMEN: Ominous Sensations	2	22.0	19.1 – 25.0	7.8	53.2	29.0
CONTA: Contagion	3	9.5	3.4 – 19.6	34.3	29.9	16.6
RETA: Accidental Mystical Retribution	5	8.8	3.6 – 18.7	24.6	42.4	16.2
RETN: Non-accidental Mystical Retribution	12	16.2	7.9 – 29.1	12.8	57.0	23.0
*Animistic/Magical Causation*						
MAGIC: Animistic/Magical	9	15.5	8.8 – 12.2	11.1	42.9	33.2
**METAPHYSICAL CAUSATION**						
META: Metaphysical	5	47.1	37.8 – 51.8	66.0	2.7	25.7

### Causal beliefs and illness types

There was variation in causal beliefs by broad illness type (Somatic (S), Mental (M), or Mental and Somatic (MS)). For example, as shown in Table 3, 69 percent of the sample that believed in the causal type of Infection/Infestation considered such causes to be associated with Somatic (S) illness compared with 23 percent believing Infection/Infestation to be a cause of both Mental and Somatic (MS) illness. Broadly, as shown in Table 3, Infection/Infestation, Physical Stress, and Organic Deterioration were more often seen as causes of Somatic (S) illness than of Mental (M) illness. On the other hand Psychological Stress and Life Events/Circumstances Stress were seen as causes of Mental (M) illness more often than of Somatic (S) illness. A mixture of causes (termed Accident Plus) including accidents, human aggression, inheritance and constitution, were seen to be predominantly causes of both Mental and Somatic (MS) illness, followed by Somatic (S) illness. Among Supernatural causes, there were no beliefs strictly associated with Somatic (S) illness. Generally, the data shown in Table 3 suggest that Supernatural beliefs were more often associated with Mental (M) illness than with Somatic (S) illness, particularly beliefs of Ominous Sensations, Mystical Retribution (both Accidental and non-Accidental), and Animistic/Mystical causes. Beliefs in Fate were associated with both Mental and Somatic (S) illness. Metaphysical beliefs, as indicated in Table 3, were associated mostly with Somatic (S) illness.

**Figure 1:**
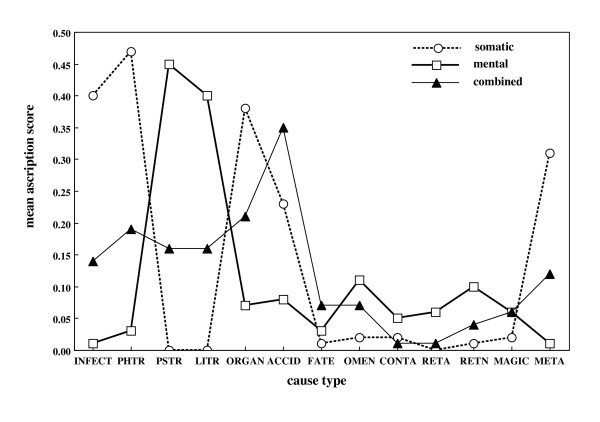
**Profile of causal beliefs**. Profile of causal beliefs as they are related by the sample to Somatic (S), Mental (M) and combined Mental and Somatic (MS) illness (INFECT = Infection/Infestation; PHTR = Physical Stress; PSTR = Psychological stress; LITR = Life Events/Circumstances Stress; ORGAN = Organic Deterioration; ACCID = Accident Plus; OMEN = Ominous Sensations; CONTA = Contagion; RETA = accidental Retribution; RETN = non-accidental Retribution; META = Metaphysical).

We carried out a further examination of the linkage of causal beliefs with illness types by developing individual scores of these associations. This was done by averaging for each participant his/her Somatic (S) illness, Mental (M) illness and Mental and Somatic (MS) ascriptions within each of the types of causal belief. Mean sample scores were then plotted (Figure 1) to give a profile of the various associations. Infection/Infestation, Physical Stress, Organic Deterioration, and Accidents Plus were most closely associated with Somatic (S) illness (Figure 1). In addition, Metaphysical Causation was associated strongly with Somatic (S) illness. Figure 1 also indicates that Psychological and Life Events/Circumstances Stress beliefs are considered as causes of Mental (M) illness. Supernatural beliefs, while relatively uncommon, are associated more closely with Mental (M) than with Somatic (S) illness. Finally most of the Natural causes featured strongly in causing both Mental and Somatic (SM) illness. These impressions were examined using a series of repeated measures analyses of variance, comparing Somatic (S), Mental and Somatic (MS) and Mental (M) illness scores for each cause type. Further examination of significance of differences was through paired t-tests. As shown in Table 4, all main effects of belief were significant and most of the specific differences between scales indicated in Figure 1 were significant with the exception of two comparisons: for Contagion, S versus SM, and for Magic M versus SM.

**Table 4:** Summary of scale endorsement differences relating to Figure 1

	F-values (df = 2, 866)	t-values (df = 443)		
		
	Belief Main Effect	Somatic vs Mental	Somatic vs Mental & Somatic	Mental vs Mental & Somatic
INFECT: Infection/Infestation	271.00^3^	24.83^3^	11.78^3^	11.21^3^
PHTR: Physical Stress	454.58^3^	34.77^3^	14.15^3^	15.65^3^
PSTR: Psychological Stress	660.39^3^	34.67^3^	20.90^3^	18.55^3^
LITR: Life Events/Circumstances Stress	549.54^3^	32.80^3^	19.49^3^	16.10^3^
ORGAN: Organic Deterioration	442.00^3^	27.19^3^	10.30^3^	14.70^3^
ACCID: Accident Plus	238.49^3^	17.63^3^	6.93^3^	23.93^3^

FATE	49.29^3^	4.28^3^	9.31^3^	5.62^3^
OMEN: Ominous Sensations	26.30^2^	7.26^3^	4.84^3^	2.90^2^
CONTA: Contagion	12.64^2^	3.27^2^	1.58^ns^	4.69^2^
RETA: Accidental Mystical Retribution	50.05^3^	9.21^2^	2.51^1^	6.30^2^
RETN: Non-accidental Mystical Retribution	88.27^3^	12.09^3^	7.71^3^	5.72^3^
MAGIC: Animistic/Magical	30.31^3^	7.18^3^	6.77^1^	< 1.00^ns^

META: Metaphysical	161.48^3^	18.07^3^	9.61^3^	9.00^3^

^ns^not significant;^1^p < .05; ^2^p < .01; ^3^p < .001

### Causal beliefs and sample diversity

Table 5 summarizes the correlations between personal characteristics and endorsement of the various causal belief types. Female gender was correlated with Supernatural beliefs, and in particular with beliefs in Fate, Ominous Sensations and Magic. Age was not correlated with any of the overall scores on Natural, Supernatural and Metaphysical beliefs. However, older participants more commonly endorsed beliefs in Fate, non-accidental Retribution and Magical causation than did younger participants. Younger participants tended to endorse Contagion beliefs more than did older participants. Marital status was correlated with the general scores on Supernatural and Metaphysical beliefs, with subsequent analyses of variance indicating those widowed or separated endorsing Supernatural beliefs (F = 3.50, df = 2, 441, p < .05) more highly than did the never married and the married. In addition, similar analyses indicated a tendency for higher endorsement of metaphysical beliefs by those who were widowed or divorced compared with the never married (F = 2.73, df = 2, 441, p = .06). Higher supernatural beliefs in those widowed or separated were found in relation to specific types including Fate, Ominous Sensations and Magical causation.

**Table 5:** Correlations between belief type scores and indicators of sample heterogeneity

	Gender	Age	Mar Stat	Educat	Urban Origin	Satisf Aust	Arriv Cohort	Accult	Eng Prof	Health	Invalid	GHQ Sympts
INFECT: Infection/Infestation	-.08	-.**09**^1^	-.03	.**17**^2^	.**11**^1^	-.**15**^2^	.**17**^2^	.**13**^2^	-.03	-.01	-.**17**^2^	-.02
PHTR: Physical Stress	-.02	.00	.03	.**14**^2^	.**16**^2^	-.**12**^1^	.**12**^2^	.**10**^1^	-.03	.05	-.07	.05
PSTR: Psychological Stress	.00	.00	.05	.**15**^2^	.**12**^1^	-.**16**^2^	.08	.**13**^2^	-.01	.09	-.03	.**13**^2^
LITR: Life Events/Circumstances Stress	-.02	.04	.08	.**16**^2^	.**17**^2^	-.**13**^1^	.08	.**13**^2^	.02	.**10**^1^	.00	.11^1^
ORGAN: Organic Deterioration	.04	-.04	.08	.**21**^2^	.**17**^2^	-.**14**^2^	.**13**^2^	.**17**^2^	.03	.03	-.**10**^1^	.**09**^1^
ACCID: Accident Plus	-.04	-.02	.05	.**16**^2^	.**21**^2^	-.**15**^2^	.**16**^2^	.**12**^1^	-.07	.02	-.05	.02

FATE	.**11**^1^	.**17**^2^	.**11**^1^	-.**22**^2^	.01	-.08	-.**10**^1^	-.**16**^2^	-.09	.**20**^2^	.**18**^2^	.**18**^2^
OMEN: Ominous Sensations	.**17**^2^	.03	.**13**^2^	-.**11**^1^	-.04	-.08	-.05	-.**11**^1^	-.**10**^1^	.**18**^2^	.07	.**26**^2^
CONTA: Contagion	.04	-.**12**^1^	-.03	.04	.04	.07	.02	.**11**^1^	.**11**^1^	-.04	.00	-.03
RETA: Accidental Mystical Retribution	.04	.05	.03	-.05	-.06	-.02	-.02	-.04	-.04	.08	.01	.08
RETN: Non-accidental Mystical Retribution	.03	.**12**^1^	.08	-.05	-.**10**^1^	-.**10**^1^	-.01	-.08	-.08	.**10**^1^	.05	.**12**^1^
MAGIC: Animistic/Magical	.**17**^2^	.**11**^1^	.**14**^2^	-.**20**^2^	-.**11**^1^	-.**10**^1^	-.04	-.**13**^2^	.**13**^2^	.**21**^2^	.**14**^2^	.**10**^1^

NATURAL	-.03	-.02	.05	.**19**^2^	.**18**^2^	-.**17**^2^	.**15**^2^	.**15**^2^	-.02	.05	-.08	.08
SUPERNAT	.**15**^2^	.08	.**12**^1^	-.**14**^2^	-.07	-.08	-.05	-.**11**^1^	-.06	.**19**^2^	.**11**^1^	.**20**^2^
META	.00	.06	.**11**^1^	.**10**^1^	.**12**^2^	-.03	-.03	.05	.06	.**10**^1^	-.02	.**13**^2^

^1^p < .05; ^2^p < .01; *degrees of freedom *range between 439 and 443 depending on missing values

NOTE: higher scores for all belief scale reflect higher endorsement of items;

*Abbreviations*: NATURAL = sum of natural types; SUPERNAT = sum of supernatural types; META = sum of metaphysical scales; Gender (female); Age (older); Mar Stat (Marital Status: divorced/widowed/separated); Educat (Education: higher education); Arriv Cohort (Arrival period cohort: recent arrival); Satisf Aust (Satisfaction with Australia: higher satisfaction); Accult (Acculturation: higher acculturation score); Eng Prof (English Proficiency: higher proficiency); Health (Self reported health: presence of illness; Invalid (Invalid status: invalidity; GHQ Sympts (sum of endorsed GHQ-60 symptoms: more symptoms)

Level of education was positively correlated with Metaphysical beliefs and negatively correlated with Supernatural beliefs. As shown in Table 5, all specific natural beliefs were associated with higher education. Additionally, the more educated were less likely to endorse the specific belief types of Fate, Ominous Sensations and Magical causation. Those originating from urban settings in Turkey were more likely than participants of rural origin to endorse all Natural and Metaphysical beliefs (Table 5). Urban origin was also associated with lower endorsement of non-Accidental Retribution and Magical causation.

With respect to year of arrival in Australia, those arriving more recently tended to endorse Natural beliefs but were no different on Supernatural and Metaphysical general scores than those arriving earlier. Recent arrival was associated with higher endorsement of Infection/Infestation, Physical Stress, Organic Deterioration and Accident Plus. There was a tendency for recent arrival to be related to lower endorsement of Fate. Acculturation level was positively associated with Natural and negatively associated with Supernatural beliefs and, indeed, all specific Natural types were correlated with acculturation (Table 5). The more acculturated were less likely to endorse the specific Supernatural beliefs of Fate, Ominous Sensation and Magic although they tended to endorse Contagion beliefs. English language proficiency was not correlated with any of the broad scales, Natural, Supernatural or Metaphysical, and only some of the specific Supernatural beliefs scales were associated with this variable including Ominous Sensations, Contagion and Magic. Satisfaction with life in Australia was negatively associated with Natural beliefs in general but uncorrelated with Supernatural and Metaphysical. Correlations with all specific Natural beliefs were significant (Table 5). Those satisfied with Australia were also less likely to endorse non-accidental Retribution and Magical causation.

### Causal beliefs and current illness

Turning to the three variables representing health status, self-reported illness and number of GHQ symptoms were correlated with Supernatural and Metaphysical beliefs while invalid status (inability to work as a result of illness) was correlated with Supernatural beliefs. Despite the lack of correlation of Natural causation with self-reported illness there were small correlations between Psychological Stress and Life Events/Circumstances Stress with self-reported illness. These belief types were also correlated with psychological symptoms measured by the GHQ, in addition to a significant association between GHQ symptoms and beliefs in Organic Deterioration. Particular associations between specific Supernatural belief types and self-reported illness included significant correlations with Fate, Ominous Sensations, non-accidental Retribution and Magical causation. The same associations proved significant in relation to psychological symptoms as measured by the GHQ. Invalid status was associated with causal types of Fate and Magic.

## Discussion

Illness causal beliefs shape illness experience, and are important in decisions about treatment choice and treatment adherence, and in the success of the therapeutic relationship. In immigrant and modernizing communities casual beliefs are subject to evolution as a result of social transactions in a changed cultural environment. In this study we examined one of the most comprehensive accounts of illness causal beliefs – proposed by Murdock et al. [7] – expanded to incorporate 'metaphysical' [2,4]. Exploratory principal components analysis revealed two broad factors accounting for 58 percent of the variation in illness belief scales. The factors were distinctly interpretable as the Murdock et al. [7] categories of natural and supernatural beliefs. We explored also where metaphysical beliefs would be placed in this sample of Turkish immigrants. The results showed that metaphysical causal beliefs were correlated most strongly with natural causal beliefs and, consistent with previous research [4], among natural causes, most strongly correlated with beliefs in Organic Deterioration and Physical Stress. As Eisenbruch [2] pointed out these can be considered natural beliefs based on a non-Western 'physiological' theory. In the Turkish immigrant sample, metaphysical causes, in addition to clustering together with natural beliefs, followed a similar pattern to somatic natural causes in correlations with other factors and in the closeness of association with somatic (S), mental (M), and both mental and somatic (MS) illness. This suggests that metaphysical causes, in the Turkish group studied, are embedded within a somatic construct of illness.

As expected, beliefs in natural causes were more frequent than beliefs in supernatural causes. The lower frequency of supernatural beliefs was not surprising given modernizing and acculturative trends and in view of the legacy of traditional beliefs described earlier [e.g. [29,34]]. This is in line with previous research within Turkey and in a small study examining Turkish beliefs in general practice and mental health service patients [18] in Melbourne. The belief in metaphysical causes by between 38 and 52 percent of the sample was somewhat surprising since none of the previous descriptions of illness representations of Turkish groups have alluded to such beliefs. Further research is required to trace the origins of such beliefs in Turks given their relatively high frequency. Among the supernatural beliefs, the most common were Ominous Sensations, non-accidental Mystical Retribution, Animistic/Magical causation and Fate. These are understandable in view of traditional beliefs described earlier. For example, non-accidental Mystical Retribution contains items that reflect the breaking of taboos, in particular non-adherence to religious and cultural practices. Some examples from our questionnaire include ingesting food or drink forbidden by religion, breaking rules considered sacred or important by one's religion, and failing to be clean or pure in one's body, mind or spirit when participating in religious ceremonies. The present findings converge with those of previous work [24-26] in Turkey in finding a high frequency of natural beliefs. In addition, it is evident from the results that the three broad belief scales were correlated with each other, consistent with previous observations that even contradictory beliefs may coexist. It is not unusual for example for patients to use multiple forms of treatment deriving from quite divergent and often incompatible explanatory models [35].

An important addition by the present study is the demonstration of both an association of particular causal beliefs with broad illness types (somatic or mental) and of causal beliefs that are not specific to illness type. This issue has not been addressed in previous research with Turkish samples, with accounts of traditional beliefs focusing on the broader construct of illness-related beliefs [29,34] or examining beliefs only in relation to mental disorders [24,25]. The results indicate that the admixture of beliefs we have labeled Accident Plus, incorporating causal beliefs such as accidents, human aggression, constitution, inheritance, together with Fate (including beliefs in destiny, luck and God's will) are thought to be more universal causes of illness. Natural causes were associated with somatic illness (infection/infestation, physical stress and organic deterioration) and with mental illness (psychological stress and life events/circumstances stress).

The last main finding is the heterogeneity within the Turkish community in the causal beliefs that are held by members of the community. A wide variety of factors were associated with variation in causal beliefs. These correlations suggest, as noted elsewhere [3,36,37], that beliefs concerning causes of illness are dynamic, determined by multiple factors, and subject to the impact of many kinds of experiences. It is clear from the findings that any assumption of uniformity of illness beliefs within a community, even one that is relatively culturally homogeneous, is likely to be misleading. From the present findings, it appears that demographic, modernizing, and acculturative factors, and the presence of illness, all contribute to the diversity of illness causal beliefs within communities. Traditional beliefs persist to varying extents despite modernizing and acculturative trends [1,35], suggesting the need to identify more clearly the conditions under which persistence and change occur. Multiple illness causal beliefs may be acquired through social and cultural exposure and by experiencing illness and contact with medical systems. Multiple causal beliefs may be held concurrently despite logical incongruities. As suggested by Patel et al. [5], rather than a competitive model, where traditional illness causal beliefs are displaced by those aligned with modern medicine, it is also possible that a cumulative model could operate, where multiple and more tentative representations of disease are present allowing much greater flexibility for remedial actions to be taken, including the use of multiple forms of treatment (e.g., complementary, traditional and orthodox medicine).

Two areas where illness causal beliefs are likely to be particularly important are in influencing pathways to care and in choice of treatment. For the latter, discrepant beliefs concerning the causes of illness between patients and clinicians may reduce the likelihood of treatment adherence [3,38,39] and contribute to poorer treatment outcomes. There is a continuing need for research to explore how disparities in illness representations between patients and clinicians may be addressed, and the effectiveness of various strategies. The task of facilitating pathways to care, and the related problem of addressing unmet need for clinical care, is a more complex one for it involves community-wide interventions. Health promotion and early intervention programs, for example, rarely incorporate culturally-informed strategies that take into account disparities regarding the health problem between professional representations and the causal beliefs of minority communities. For mental disorders, where there may be a substantial disparity between professional and community representations [e.g. [19,40-42]], professional explanations of causes of illness may be seen by members of minority communities as inappropriate, confusing or irrelevant.

## Conclusion

There is growing research attention to illness representations. A better understanding of the diversity, and determinants, of such representations can be of value in improving our understanding of illness experience and the clinical process, and in developing more effective population health strategies. In this study causal beliefs of a Turkish immigrant community were assessed for the relative frequency of natural, supernatural and metaphysical beliefs, showing persistence of supernatural beliefs as well as a high frequency of natural beliefs. A small but significant number of participants also endorsed metaphysical beliefs. Several belief types are regarded as causing both mental and somatic illness while others are seen as more likely to cause either somatic or mental illness. Finally the study showed that causal beliefs are subject to variation within the community studied, based on demographic, modernizing, and acculturative factors and the presence of illness. Improvements in cross-cultural health care may be facilitated through addressing how discrepancies between community and professional illness representations may be effectively negotiated.

## Competing interests

The author(s) declare that they have no competing interests.

## Authors' contributions

HM and SK designed the project, secured the research grant, developed research instruments. SK carried out the statistical analyses. HM and SK wrote the drafts of the paper. RK and CT contributed to the writing of the paper. CT carried out translations of research instruments from English to Turkish. SK, HM and CT trained and supervised research assistants and research interviewers. HM wrote the final draft of the paper which was approved by all authors.

## Pre-publication history

The pre-publication history for this paper can be accessed here:


